# Epigenetic downregulation of MAPKAPK2 exacerbates oxidative stress-induced damage in vitiligo melanocyte cell line model

**DOI:** 10.3389/fmed.2026.1698091

**Published:** 2026-04-20

**Authors:** Yishan Wang, Xiaoyong Zheng

**Affiliations:** Department of Dermatology, The Affiliated Taizhou People's Hospital of Nanjing Medical University, Taizhou, Jiangsu, China

**Keywords:** epigenetic regulation, KEAP1/Nrf2/HO-1 signaling, MAPKAPK2, oxidative stress, vitiligo

## Abstract

**Background and objective:**

Vitiligo is an acquired depigmentation disorder caused by melanocyte dysfunction or loss. Oxidative stress is widely considered a key driver to its pathogenesis. Mitogen-activated protein kinase-activated protein kinase 2 (MAPKAPK2) is implicated in oxidative stress responses, although its role in vitiligo remains uncertain. This study intended to investigate whether epigenetic downregulation of MAPKAPK2 aggravates oxidative stress-induced damage in vitiligo melanocytes.

**Methods:**

Human melanocyte lines (PIG1 and PIG3V) were used to model normal and vitiligo conditions. The effects of oxidative stress, DNA demethylation (5-aza-DC), and MAPKAPK2 overexpression were assessed using qRT-PCR, Western blot, ELISA, comet assay, TUNEL, and CCK-8. Pharmacological inhibition of MK2 was employed to evaluate the functional requirement of MAPKAPK2 kinase activity, and key antioxidant pathways, including Nrf2 signaling, were investigated.

**Results:**

MAPKAPK2 expression was notably downregulated in PIG3V cells compared with PIG1 cells (*P* < *0.05*), and further reduced upon H_2_O_2_ exposure (*P* < *0.01*), suggesting stress-related suppression. Exposure to 5-aza-DC partially restored MAPKAPK2 expression (*P* < *0.01*), implicating DNA methylation in its silencing. Functional assays showed that MAPKAPK2 overexpression significantly alleviated H_2_O_2_-induced reductions in cell viability, increases in apoptosis, impaired melanogenesis, and oxidative damage (all *P* < *0.01*), while activating the Nrf2/HO-1 antioxidant pathway through suppression of KEAP1 expression and enhancement of Nrf2 nuclear translocation. Genetic knockdown, rescue, and pharmacological inhibition experiments further demonstrated that these cytoprotective effects under oxidative stress were dependent on MAPKAPK2 kinase activity.

**Conclusion:**

Epigenetic silencing of MAPKAPK2 aggravates oxidative damage in vitiligo melanocytes, potentially by attenuating Nrf2-associated antioxidant responses. These findings identify MAPKAPK2 as a functionally relevant and targetable factor in the oxidative pathology of vitiligo.

## 1 Introduction

Vitiligo is a common acquired depigmentation disorder caused by the loss or dysfunction of melanocytes, leading to patchy skin depigmentation (1). Epidemiological studies estimate a global prevalence of 0.5%−2%, affecting all age groups and causing substantial psychological and social burdens (2). Although the pathogenesis of vitiligo has been extensively investigated—encompassing autoimmunity, apoptosis, genetic predisposition, and oxidative stress (3)—its exact etiology and key molecular mechanisms remain unclear. In clinical settings, treatment options are limited by high recurrence rates and variable efficacy. Clarifying key molecular mechanisms and identifying new therapeutic targets remain priorities. Oxidative stress is considered a central pathogenic mechanism in vitiligo (4). Lesional skin of vitiligo patients often shows excessive accumulation of reactive oxygen species (ROS) as well as reduced activity in antioxidant defenses (5), such as glutathione (GSH), superoxide dismutase (SOD), and catalase (CAT). Elevated ROS concentration can lead to genetic damage, oxidative lipid breakdown, and protein dysfunction in melanocytes, ultimately leading to functional impairment and apoptosis (6, 7). However, the mechanisms underlying oxidative vulnerability in vitiligo melanocytes remain unclear.

MAPKAPK2 (mitogen-activated protein kinase-activated protein kinase 2) is an essential downstream effector within the p38 MAPK signaling axis, involved in inflammatory responses, oxidative stress signaling, and DNA damage repair (8, 9). It has been shown to exert anti-apoptotic and antioxidant effects by modulating various cytoprotective pathways, particularly in inflammatory and tumor contexts (10, 11). However, its expression and functional role in melanocytes, especially under the pathological conditions of vitiligo, remain unexplored. It is therefore plausible that MAPKAPK2 plays a regulatory role in the oxidative stress response of melanocytes in vitiligo, and that its dysregulation may contribute to disease pathogenesis.

Recent advances in epigenetics have provided new insights into the molecular mechanisms of disease (12). DNA methylation, among the most common epigenetic regulatory mechanisms (13), serves an essential function in modulating gene transcription and has been linked to various skin disorders, including psoriasis and atopic dermatitis (14). In oxidative stress-related pathways, aberrant DNA methylation may exacerbate cellular damage by silencing protective genes (15). Therefore, investigating whether MAPKAPK2 is downregulated by DNA methylation and whether this regulation contributes to oxidative stress–induced dysfunction in melanocytes warrants further exploration.

In this study, we hypothesize that MAPKAPK2 is epigenetically repressed in vitiligo melanocytes, rendering them more susceptible to oxidative stress. Using normal and vitiligo-derived melanocytes, we explored the impact of oxidative stimuli, DNA demethylation, and MAPKAPK2 overexpression on redox homeostasis, melanogenic function. This work aims to determine whether MAPKAPK2 acts as a key regulator of oxidative damage and whether its downregulation involves epigenetic silencing, thereby providing mechanistic insight and a potential therapeutic target for vitiligo.

## 2 Materials and methods

### 2.1 Cell line preparation and grouping

This investigation employed two human melanocyte cell lines: PIG1 (normal human melanocytes, #YS1767C, Shanghai Yaji Biothechnology Co., Ltd., China) and PIG3V (vitiligo melanocytes, #BFN60810796, Qingqi Biotechnology, Shanghai, China) (16–18). Both cell lines were authenticated by short tandem repeat (STR) analysis and free of mycoplasma contamination. Cells were maintained in RPMI-1640 medium (Gibco, Thermo Fisher Scientific, USA) containing 10% fetal bovine serum (FBS) and 1% penicillin-streptomycin under incubation at 37 °C (humidified atmosphere containing 5% CO_2_).

To investigate the role of MAPKAPK2 under oxidative stress conditions, the PIG3V cell line was divided into six experimental groups: Control group: untreated cells; H_2_O_2_ group: treated with 1 mM hydrogen peroxide (H_2_O_2_) for 24 h; 5-aza-DC group: treated with 20 μM 5-aza2′-deoxycytidine (5-aza-DC) for 24 h to induce DNA demethylation; H_2_O_2_ + 5-aza-DC group: co-treated with 1 mM H_2_O_2_ and 20 μM 5-aza-DC for 24 h; H_2_O_2_ + vector group: transfected with the empty pcDNA3.1 vector followed by 1 mM H_2_O_2_ for 24 h; H_2_O_2_ + MAPKAPK2 group: transfected with the pcDNA3.1-MAPKAPK2 plasmid followed by 1 m MH_2_O_2_ for 24 h.

To functionally evaluate the requirement of MAPKAPK2 kinase activity, additional pharmacological inhibition groups were included: MK2 inhibitor group: treated with the selective MK2 inhibitor PF-3644022 (0.5 μM) alone without H_2_O_2_ exposure; H_2_O_2_+ MAPKAPK2 + inhibitor group: PIG3V cells were transfected with pcDNA3.1-MAPKAPK2 plasmid, pretreated with PF-3644022 for 1 h, and subsequently exposed to 1 mM H_2_O_2_ for 24 h with the inhibitor maintained throughout the treatment period.

For loss-of-function and rescue experiments, additional groups were established as follows: si-NC group, transfected with negative control siRNA; si-MAPKAPK2 group, transfected with MAPKAPK2-specific siRNA; H_2_O_2_ + si-NC group, transfected with si-NC followed by H_2_O_2_ treatment; H_2_O_2_ + si-MAPKAPK2 group, transfected with si-MAPKAPK2 followed by H_2_O_2_ treatment; and H_2_O_2_ + si-MAPKAPK2 + MAPKAPK2 group, transfected with si-MAPKAPK2 followed by re-expression of MAPKAPK2 and subsequent H_2_O_2_ treatment.

For transfection experiments, PIG3V cells were seeded in six-well plates and grown to approximately 60%−70% confluence. siRNA transfection was first performed using Lipofectamine™ 3000 (Invitrogen, Thermo Fisher Scientific, USA) according to the manufacturer's instructions. After 24 h of siRNA transfection, cells were subjected to plasmid transfection with either pcDNA3.1 empty vector or pcDNA3.1-MAPKAPK2 plasmid using the same transfection reagent. Briefly, plasmids were diluted in Opti-MEM medium and mixed with Lipofectamine™ 3000 reagent before being added to the cells. After 6 h of plasmid transfection, the medium was replaced with fresh complete RPMI-1640 medium to minimize cytotoxicity, and cells were allowed to recover for an additional 24 h to ensure sufficient MAPKAPK2 expression. Following the recovery period, transfected cells were exposed to 1 mM H_2_O_2_ for 24 h, after which cells were harvested for downstream analyses, including qRT-PCR, Western blotting, cell viability assays, and oxidative stress–related functional assays, as indicated.

The concentration and exposure duration of H_2_O_2_ were chosen based on previous studies demonstrating that 1 mM H_2_O_2_ for 24 h effectively induces oxidative stress while maintaining acceptable cell viability in melanocyte models of vitiligo (16–18), thus providing a reproducible *in vitro* system to mimic oxidative injury. In addition, to assess the dose-dependent effect of oxidative stress on MAPKAPK2 expression, PIG3V cells were separately exposed to increasing concentrations of H_2_O_2_ (0.25, 0.5, and 1.0 mM) for 24 h, with untreated cells serving as controls.

### 2.2 Cell viability assay

PIG3V cells from the Control, H_2_O_2_, 5-aza-DC, H_2_O_2_ + 5-aza-DC, H_2_O_2_ + vector, H_2_O_2_ + MAPKAPK2, MK2 inhibitor, and H_2_O_2_ + MAPKAPK2 + inhibitor groups were evaluated for viability with the Cell Counting Kit-8 (CCK-8, #C0037, Beyotime, China), according to the supplier's instructions. Cells were plated at 5 × 103 cells per well in 96-well plates, with 100 μL complete RPMI-1640, then permitted to adhere during the night. After corresponding treatments, 10 μL of CCK-8 was applied and kept at 37 °C for 2 h. Optical density (OD450) was detected by a microplate reader (BioTek Synergy H1, USA), and viability was calculated as a percentage standardized against the Control group. Each assay was conducted three times independently.

### 2.3 TUNEL assay

TUNEL staining was conducted on PIG3V cells from the Control, H_2_O_2_, H_2_O_2_ + vector, and H_2_O_2_ + MAPKAPK2 groups via the One Step TUNEL Apoptosis Assay Kit (#C1088), 0.3% Triton X-100 (#P0096), and DAPI (all from Beyotime, China). Cells were cultured on glass coverslips, treated with 4% paraformaldehyde for 30 min, rendered permeable with 0.3% Triton X-100 (5 min), and exposed to TUNEL assay reagent at 37 °C for 1 h under dark conditions. Counterstaining of nuclei was completed with DAPI for fluorescence visualization.

Images were captured through an Olympus IX73 platform, and apoptotic cells were evaluated as the ratio of TUNEL-positive (green) nuclei out of all DAPI-labeled (blue) nuclei in five random fields per coverslip. Each experiment was conducted in triplicate.

### 2.4 Enzyme-linked immunosorbent assay (ELISA)

PIG3V cells from the Control, H_2_O_2_, H_2_O_2_ + vector, and H_2_O_2_ + MAPKAPK2 groups were harvested after treatment. For melanin quantification, cells were lysed using 1 mol/L NaOH at 80 °C for 1 h, and OD405 was read with a BioTek Synergy H1 plate reader (USA). Tyrosinase activity as well as 8-hydroxy-2′-deoxyguanosine (8-OHdG) concentrations were quantified by means of ELISA kits (Elabscience, China): Tyrosinase Activity Assay Kit (#E-EL-M0753c) and 8-OHdG ELISA Kit (#E-EL-0028). All procedures followed the manufacturer's instructions. Protein concentrations were normalized using a BCA assay (Beyotime, China). Assays were independently repeated three times.

### 2.5 Measurement of oxidative stress indicators using commercial kits

ROS, MDA, GSH, and SOD activity were measured in PIG3V cells from the Control, H_2_O_2_, H_2_O_2_ + vector, and H_2_O_2_ + MAPKAPK2 groups using analytical kits from Nanjing Jiancheng Bioengineering Institute (China). ROS was assessed using the DCFH-DA fluorescent probe (#E004-1-1) by exposing cells to 10 μM DCFH-DA at 37 °C for 30 min in the absence of light. Fluorescent signals were visualized with an Olympus IX73 microscopic system and quantified with ImageJ software. MDA (#A003-1-2), GSH (#A006-2-1), as well as SOD (#A001-3-2) levels were determined by colorimetric kits as instructed by the manufacturer's protocols. All results were standardized based on total protein content and expressed as mean values from triplicate experiments.

### 2.6 Comet assay

DNA damage was assessed in PIG3V cells from the Control, H_2_O_2_, H_2_O_2_ + vector, and H_2_O_2_ + MAPKAPK2 groups using the alkaline comet assay. Cells were embedded in low-temperature agarose matrices on comet slides, lysed in alkaline cell lysis solution (pH 10) at 4 °C for 1 h, and then incubated in alkaline unwinding buffer (pH >13) for 30 min. An electrophoretic run was carried out at 25 V and 300 mA for 30 min. After neutralization, slides received propidium iodide staining (2 μg/ml) and were viewed under a fluorescence microscope (Olympus IX73, Japan). At least 50 cells per group were analyzed with Comet Assay Software Project (CASP), and DNA injury was quantified by tail DNA% and tail moment. All experiments were performed in triplicate.

### 2.7 RNA extraction and quantitative real-time PCR (qRT-PCR)

Total RNA was extracted from PIG1 and PIG3V cells using TRIzol™ reagent (#15596018, Invitrogen, USA), and its concentration and purity were assessed using a NanoDrop™ 2000 device (Thermo Fisher, USA). Samples with RNA purity (A260/A280 within 1.8 and 2.0) were selected for cDNA synthesis via the PrimeScript™ RT reagent kit with gDNA Eraser (#RR047A, Takara, Japan), incorporating 1 μg RNA in 20 μL mixture. TB Green^®^ Premix Ex Taq™ II (#RR820A, Takara, Japan) was applied for qRT-PCR on the Applied Biosystems StepOnePlus™ PCR machine (USA). The primer sequences for MAPKAPK2 were: Forward: 5′-ACAAAGGTCCCTCAAACCCC-3′; Reverse: 5′-ATCCTCTGCTCACAACCTGG-3′. GAPDH served as the reference gene, and relative mRNA expression was calculated via the 2^−Δ*ΔCt*^ approach. Each measurement was repeated three times.

### 2.8 Immunoblot analysis

Total protein was isolated from PIG1 and PIG3V cells disrupted in RIPA lysis buffer (Beyotime, China) with protease and phosphatase inhibitor cocktails, and quantified via BCA assay (Beyotime, China). 30 μg per lane was loaded onto 10%−12% SDS-PAGE gels and transferred to PVDF membranes (Millipore, USA). Membranes were pretreated with 5% non-fat milk/TBST (1 h) before overnight exposure at 4 °C to antibodies targeting MAPKAPK2 (ab32567), HO-1 (ab189491), KEAP1 (ab119403), Nrf2 (ab137550), γ-H2AX (ab81299), PARP-1 (ab191217), CHK2 (ab207446), phosphorylated CHK2 (p-CHK2, ab59408), TYR (ab170905), PMEL (ab137078), MITF (ab12039), p-HSP27 (ab155987), HSP27 (ab12351), β-actin (ab8226), and Histone H3 (ab1791) (all from Abcam, UK). All primary antibodies were used at a dilution of 1:1,000, except β-actin (1:5,000). Following incubation with HRP- labeled secondary antibodies (1:5,000, Abcam, UK), signals were revealed via enhanced chemiluminescence (ECL) reagents (Millipore, USA) and captured with a ChemiDoc™ MP platform (Bio-Rad, USA).

For subcellular localization of Nrf2, cytosolic and nuclear protein fractions were prepared using a commercial nuclear extraction kit (NE-PER^™^, Thermo Fisher Scientific) according to the manufacturer's instructions. Protein band intensities were quantified using ImageJ software (NIH, USA) after background subtraction and normalized to β-actin (for total and cytoplasmic proteins) or Histone H3 (for nuclear proteins).

### 2.9 Statistical analysis

SPSS software (v23.0; IBM, Armonk, NY) was utilized for statistical analysis. Results are reported as mean ± standard deviation (SD) from at least three biological replicates. Group-level analysis was performed via one-way analysis of variance (ANOVA) and subsequent Tukey *post hoc* analysis. Differences with *p* < 0.05 were deemed statistically significant.

## 3 Results

### 3.1 Basal epigenetic repression and oxidative stress–associated suppression of MAPKAPK2 in vitiligo melanocytes

**Figure 1:**
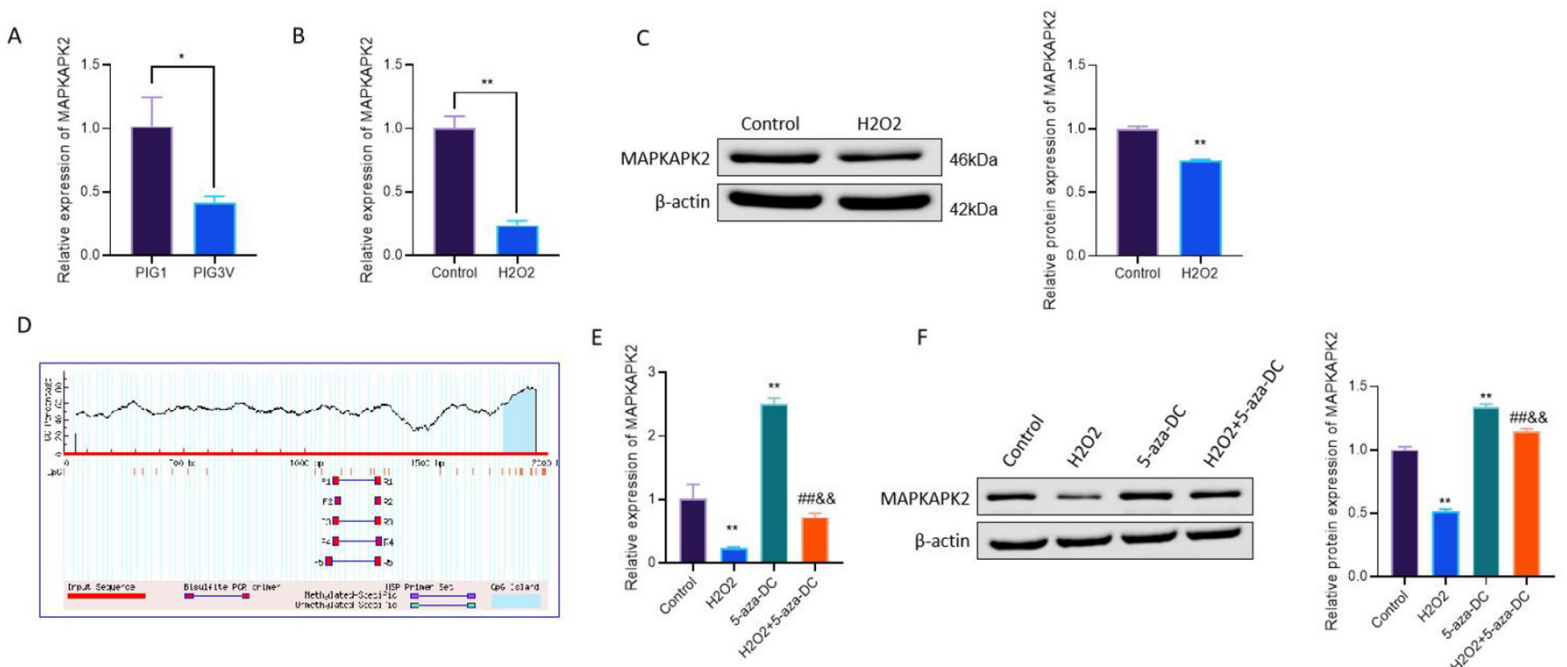
MAPKAPK2 expression is suppressed in vitiligo melanocytes and modulated by oxidative stress and DNA methylation. **(A, B)** qRT-PCR measurement of MAPKAPK2 mRNA expression in PIG1 vs. PIG3V cells and in PIG3V cells ± H_2_O_2_. **(C)** Immunoblotting of MAPKAPK2 protein expression after H_2_O_2_ treatment. **(D)** CpG mapping prediction within MAPKAPK2 promoter. **(E, F)** MAPKAPK2 transcript and protein levels in PIG3V cells administered 5-aza-DC ± H_2_O_2_.*P < 0.05, **P < 0.01 vs. PIG1 or Control; ^##^*P* < 0.01 vs. H_2_O_2_; ^&&^*P* < 0.01 vs. 5-aza-DC.

To investigate the expression of MAPKAPK2 in vitiligo melanocytes, we first compared its levels between PIG1 and PIG3V cells. qRT-PCR assays revealed a pronounced downregulation in MAPKAPK2 mRNA content in PIG3V cells relative to PIG1 (*P* < 0.05; Figure 1A), indicating basal repression of MAPKAPK2 in vitiligo melanocytes. To assess the effect of oxidative stress on MAPKAPK2 expression, PIG3V cells were exposed to increasing concentrations of H_2_O_2_. MAPKAPK2 mRNA levels exhibited a dose-dependent decrease following H_2_O_2_ treatment (*P* < 0.001; Supplementary Figure S1A). This trend was further confirmed at the protein level by Western blot analysis, which showed a corresponding reduction in MAPKAPK2 protein abundance with increasing H_2_O_2_ concentrations (Supplementary Figure S1B). In contrast to vitiligo melanocytes, H_2_O_2_-treated PIG1 cells did not significantly alter MAPKAPK2 mRNA or protein expression in PIG1 cells (Supplementary Figures S2A, B), indicating a differential response to oxidative stress between normal and vitiligo melanocytes.

Based on this dose–response analysis, 1.0 mM H_2_O_2_ was selected as the working concentration for subsequent experiments, under which MAPKAPK2 expression was significantly reduced at both the mRNA and protein levels in PIG3V cells (*P* < 0.01; Figures 1B, C), confirming effective oxidative stress–associated suppression under the experimental conditions used thereafter. To explore whether epigenetic regulation contributes to the basal repression of MAPKAPK2, *in silico* CpG island prediction using MethPrimer identified dense CpG motifs within the MAPKAPK2 promoter region (Figure 1D), implicating potential DNA methylation–mediated regulation. Based on this observation, PIG3V cells were treated with the 5-aza-DC. Application of 5-aza-DC significantly upregulated MAPKAPK2 mRNA and protein expression under basal conditions and partially restored its expression in H_2_O_2_-treated cells (P <0.01; Figures 1E, F), suggesting that DNA methylation may contribute, at least in part, to the basal repression of MAPKAPK2 in vitiligo melanocytes.

These findings suggest that MAPKAPK2 downregulation in vitiligo melanocytes is exacerbated under oxidative stress conditions, while its basal repression can be partially reversed by targeting DNA methylation, highlighting a potential contribution of epigenetic regulation to melanocyte vulnerability.

### 3.2 MAPKAPK2 overexpression mitigates H2O2-triggered cellular toxicity as well as restores melanogenic capacity within melanocytes affected by vitiligo

**Figure 2:**
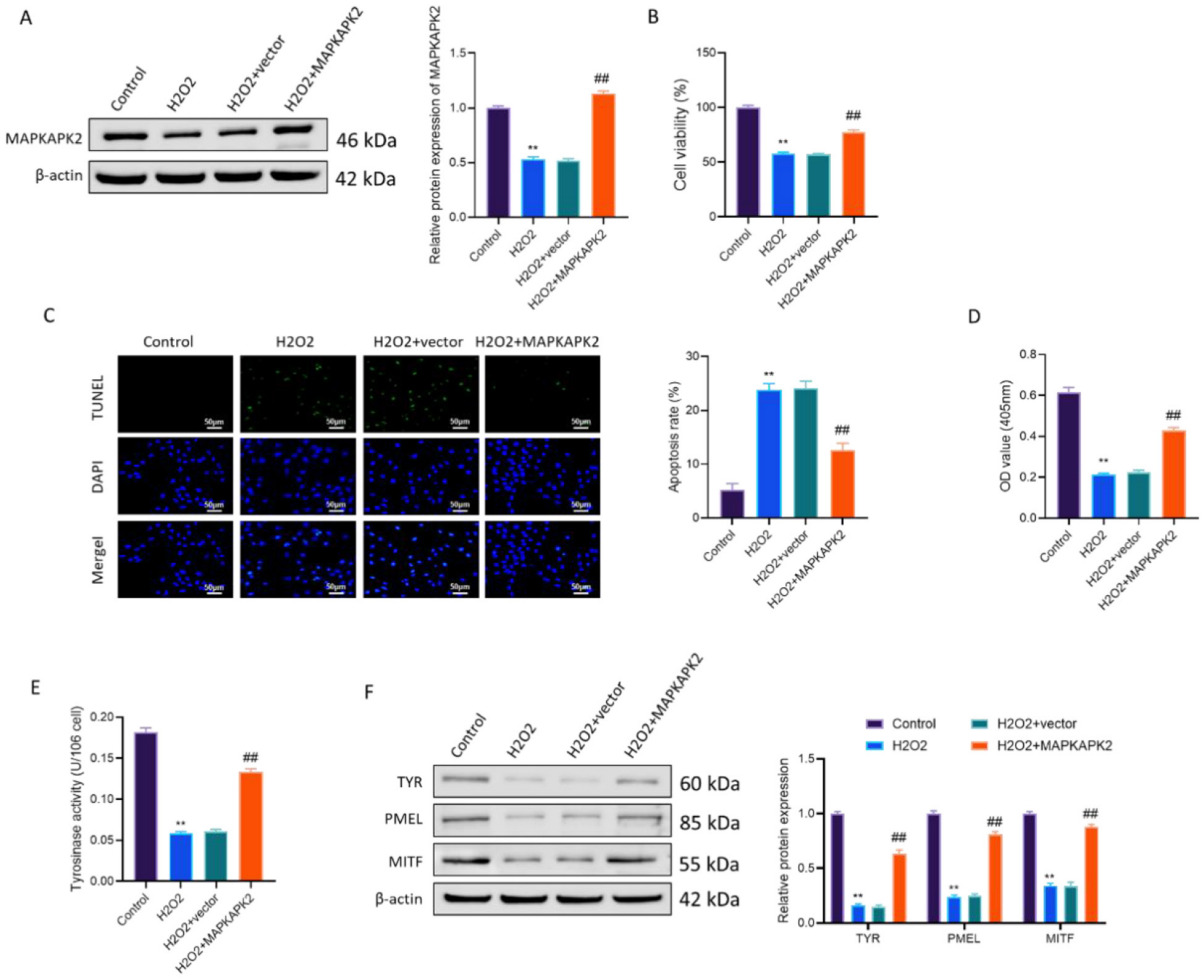
MAPKAPK2 overexpression mitigates H_2_O_2_-triggered cellular toxicity as well as restores melanogenic capacity within melanocytes affected by vitiligo. **(A)** Western blot verification of MAPKAPK2 overexpression. **(B)** CCK-8 assay–based evaluation of cell viability. **(C)** Apoptosis analysis through TUNEL staining and quantification. **(D)** Melanin content. **(E)** Tyrosinase activity. **(F)** Immunoblot analysis of TYR, PMEL, as well as MITF. **P < 0.01 vs. Control; ^##^*P* < 0.01 vs. H_2_O_2_ + vector.

To evaluate the cytoprotective function of MAPKAPK2 under oxidative insult conditions, PIG3V cells were exposed to H_2_O_2_ and transfected with either an empty vector or MAPKAPK2-expressing plasmid. Western blot confirmed successful MAPKAPK2 overexpression (Figure 2A).

Functionally, H_2_O_2_ reduced cell survival and increased apoptosis, as shown via CCK-8 and TUNEL assays, in the order presented (*P* < 0.01). MAPKAPK2 overexpression markedly restored cell viability and reduced apoptosis (*P* < 0.01), indicating cytoprotective effects (Figures 2B, C). Similarly, oxidative stress also led to decreased melanin content and suppressed tyrosinase activity (*P* < 0.01), but partially restored in MAPKAPK2-overexpressing cells (*P* < 0.01) (Figures 2D, E). Consistent with these phenotypic changes, Western blot analysis showed that H_2_O_2_ downregulated the expression of key melanogenic proteins—TYR, PMEL, and MITF (*P* < 0.01). MAPKAPK2 overexpression robustly normalized the levels of all three diagnostic markers to near-control levels (*P* < 0.01) (Figure 2F).

The above observations revealed that MAPKAPK2 could effectively counteract H_2_O_2_-induced cellular damage in vitiligo melanocytes by enhancing viability, reducing apoptosis, and restoring melanogenic function.

### 3.3 MAPKAPK2 overexpression reduces ROS accumulation and restores antioxidant capacity in H_2_O_2_-treated oxidative stress

To evaluate the antioxidant regulatory function of MAPKAPK2, we assessed intracellular ROS levels and key antioxidant markers in H_2_O_2_-treated PIG3V cells with or without MAPKAPK2 overexpression.

**Figure 3:**
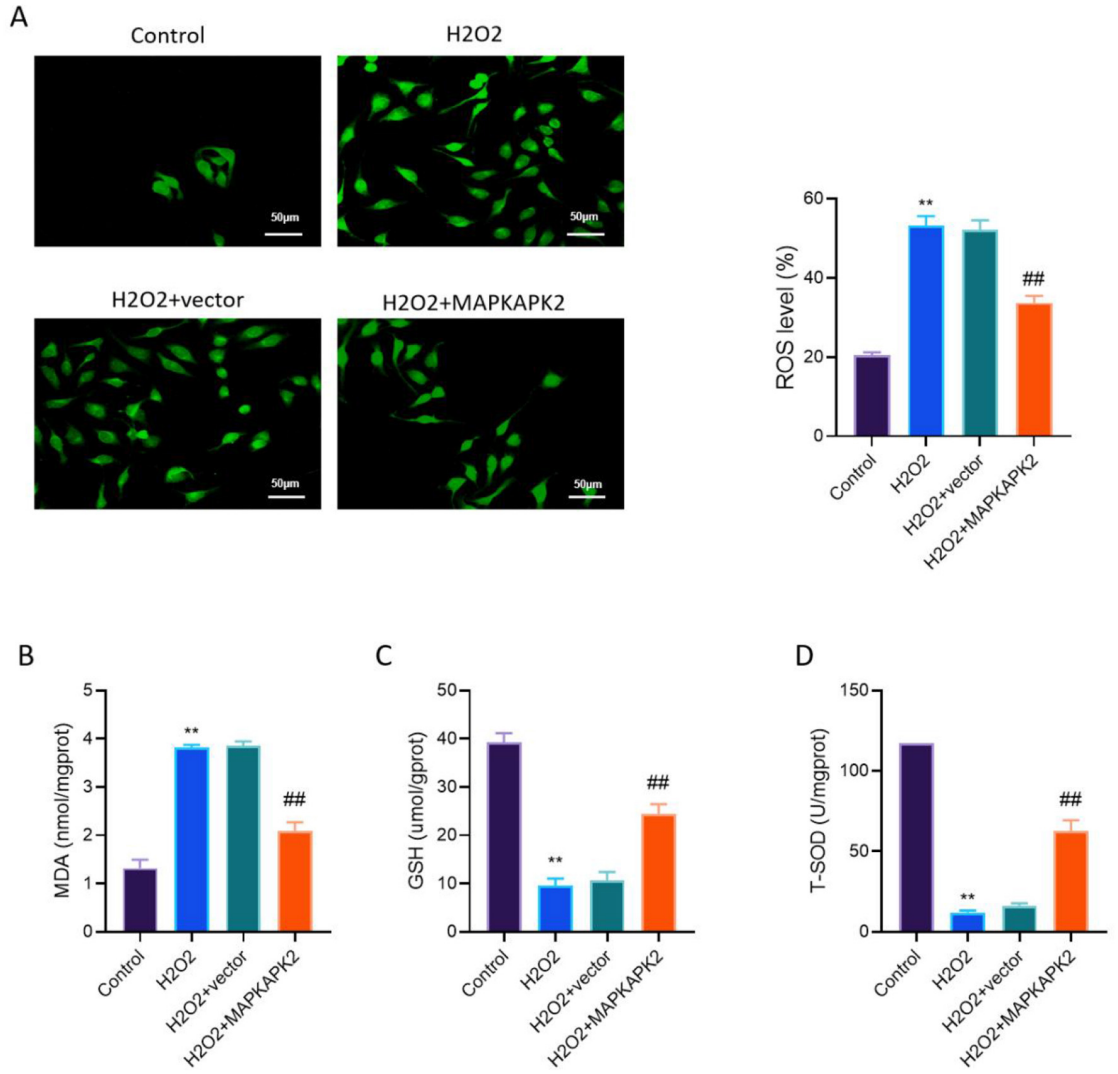
MAPKAPK2 overexpression reduces intracellular oxidative stress and restores antioxidant capacity in H_2_O_2_-treated vitiligo melanocytes. **(A)** ROS fluorescence staining and quantification. **(B)** MDA levels. **(C)** GSH content. **(D)** T-SOD activity. **P < 0.01 vs. Control; ^##^*P* < 0.01 vs. H_2_O_2_ + vector.

As shown in Figure 3A, H_2_O_2_ markedly increased ROS levels, as indicated by enhanced green fluorescence, with ROS levels increasing by approximately 2.4-fold compared with the control group, while MAPKAPK2 overexpression significantly reduced ROS accumulation by approximately 35%−40% compared with the H_2_O_2_ + vector group (*P* < 0.01). A consistent trend was observed in MDA levels, which were elevated under oxidative stress (approximately 3.0-fold higher than the control) but significantly decreased in the MAPKAPK2-overexpressing group by approximately 40%−45% relative to the H_2_O_2_ + vector group (Figure 3B). Conversely, antioxidant indicators such as GSH and T-SOD showed sharp declines following H_2_O_2_ exposure. GSH levels decreased by approximately 70% compared with the control group, whereas MAPKAPK2 overexpression increased GSH levels by approximately 2.5-fold relative to the H_2_O_2_ + vector group (Figure 3C). Similarly, T-SOD activity decreased by nearly 80% after H_2_O_2_ treatment, while MAPKAPK2 overexpression restored T-SOD activity by approximately 3–4-fold compared with the H_2_O_2_ + vector group (*P* < 0.01), suggesting effective rescue of cellular antioxidant capacity (Figure 3D).

These findings indicated that MAPKAPK2 could reduce ROS production and enhance antioxidant capacity, providing phenotypic evidence that precedes further functional validation of its enzymatic activity under oxidative stress.

### 3.4 MAPKAPK2 kinase activity is required for its cytoprotective effects under oxidative stress

To determine whether the antioxidant and cytoprotective effects observed above are dependent on MAPKAPK2 enzymatic activity rather than protein abundance alone, giving that MAPKAPK2 is a serine/threonine kinase, changes in its expression alone may not necessarily reflect functional activation. Therefore, we next examined whether the cytoprotective effects of MAPKAPK2 under oxidative stress are dependent on its kinase activity by using a selective MK2 pharmacological inhibitor (PF-3644022).

**Figure 4:**
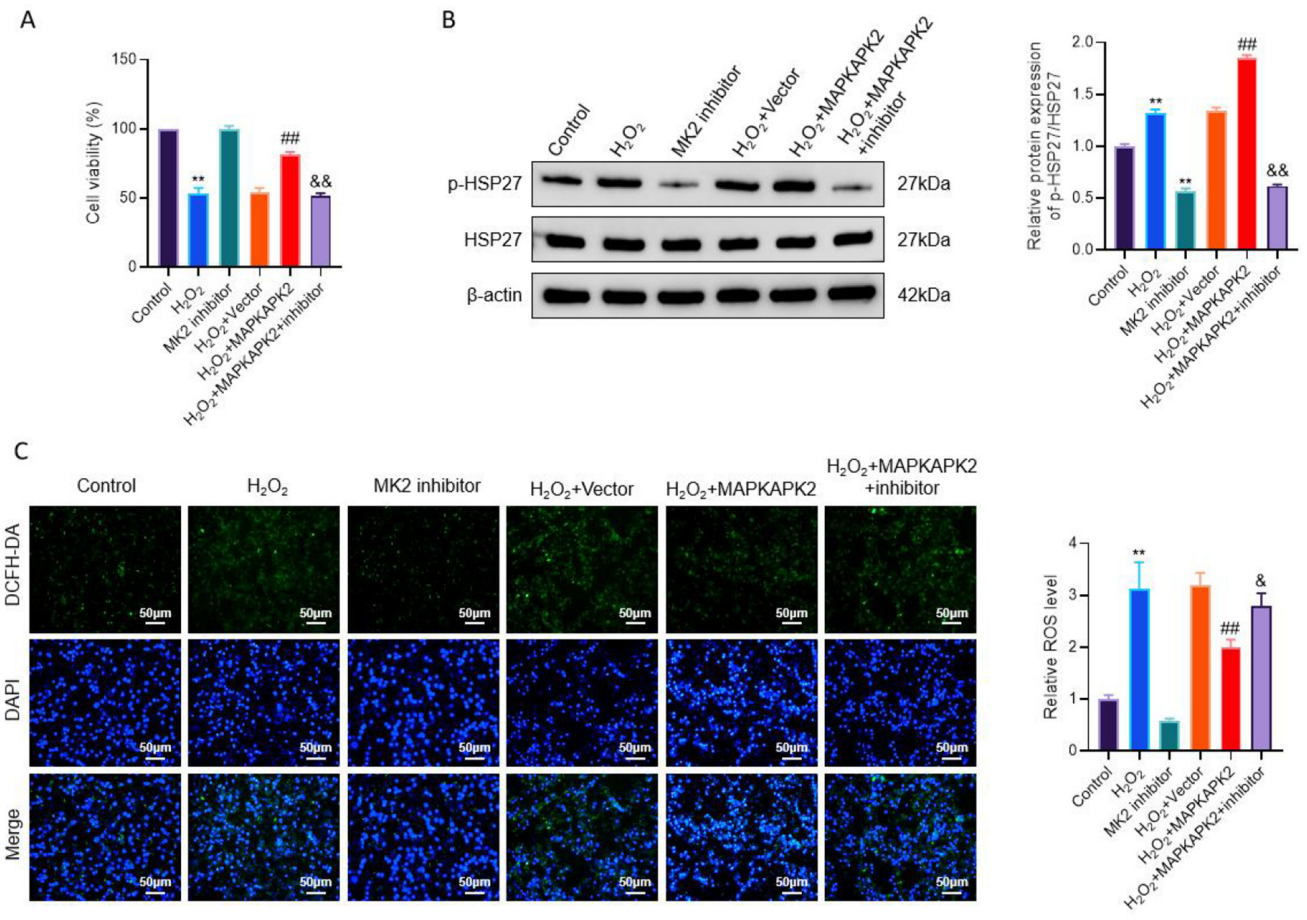
MAPKAPK2 kinase activity is essential for these cytoprotective effects under oxidative stress. **(A)** Cell viability of PIG3V cells measured by CCK-8 assay under the indicated treatments, including H_2_O_2_ exposure, MAPKAPK2 overexpression, and MK2 inhibition. **(B)** Representative Western blot images and quantitative analysis of p-HSP27 and total HSP27 protein levels. **(C)** Intracellular ROS levels detected by DCFH-DA fluorescence staining (green) with DAPI nuclear counterstaining (blue), along with corresponding quantitative analysis. **P < 0.01 vs. Control; ^##^*P* < 0.01 vs. H_2_O_2_ + vector; ^&^*P* < 0.05; ^&&^*P* < 0.01 vs. H_2_O2 + MAPKAPK2.

As shown in Figure 4A, treatment with the MK2 inhibitor alone did not result in a significant change in cell viability under basal conditions, indicating that basal survival of PIG3V cells is not critically dependent on MK2 activity. In contrast, H_2_O_2_ exposure markedly reduced cell viability, which was significantly restored by MAPKAPK2 overexpression. Importantly, co-treatment with the MK2 inhibitor largely abolished the protective effect conferred by MAPKAPK2 overexpression under oxidative stress conditions. These findings indicate that although MK2 inhibition alone is insufficient to affect basal cell viability, intact MK2 kinase activity is required for MAPKAPK2-mediated cytoprotection in response to oxidative injury. To directly assess MK2 activity, phosphorylation of HSP27, a well-established downstream substrate of MK2, was evaluated. Western blot analysis demonstrated that H_2_O_2_ treatment and MAPKAPK2 overexpression significantly increased p-HSP27 levels, whereas total HSP27 expression remained unchanged. Importantly, MK2 inhibition effectively suppressed HSP27 phosphorylation, including in MAPKAPK2-overexpressing cells, confirming successful blockade of MK2 kinase activity (Figure 4B). Consistent with the above functional and biochemical findings, intracellular ROS levels assessed by DCFH-DA fluorescence staining showed that MAPKAPK2 overexpression markedly attenuated H2O2-induced ROS accumulation, reducing ROS levels by approximately 30%−40% compared with the H_2_O_2_ + vector group. However, this antioxidant effect was significantly reversed by MK2 inhibitor treatment, which increased ROS levels by approximately 40%−50% relative to the H_2_O_2_ + MAPKAPK2 group (Figure 4C).

Collectively, these results demonstrate that the protective effects of MAPKAPK2 against oxidative stress–induced cellular injury are dependent on its kinase activity rather than merely increased protein expression, underscoring the functional importance of MK2 enzymatic activation in vitiligo melanocytes.

### 3.5 MAPKAPK2 overexpression mitigates oxidative DNA damage and suppresses DNA damage response activation in vitiligo melanocytes

To further evaluate the protective role of MAPKAPK2 in maintaining genomic stability under oxidative stress, we assessed DNA damage using comet assay, 8-OHdG quantification, and DNA damage response (DDR) protein expression.

**Figure 5:**
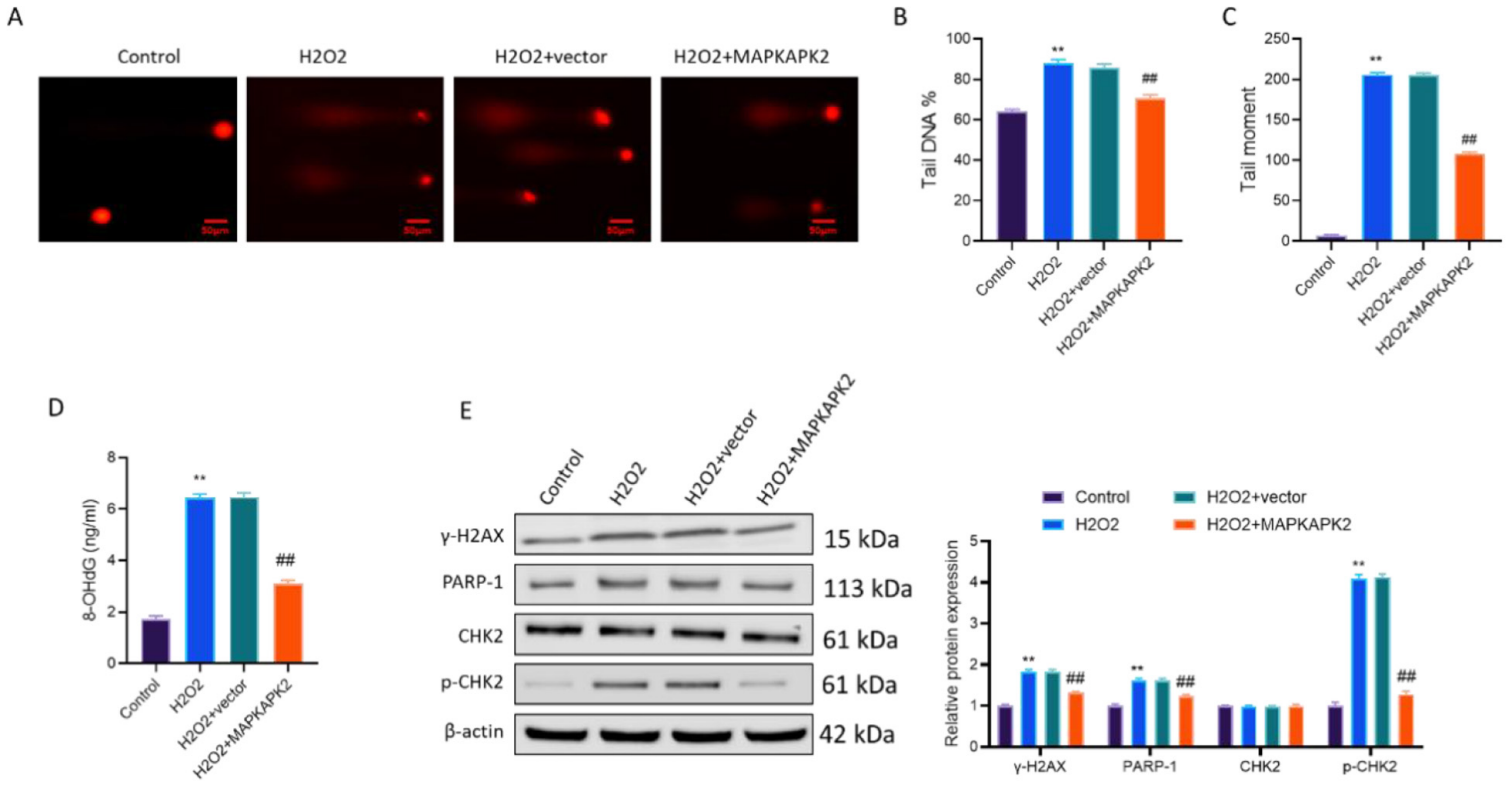
MAPKAPK2 overexpression attenuates oxidative stress-induced DNA damage and DNA damage response activation in vitiligo melanocytes. **(A)** Comet assay showing DNA fragmentation. **(B, C)** Quantification of tail DNA (%) and tail moment. **(D)** 8-OHdG levels detected by ELISA. **(E)** Immunoblotting of γ-H2AX, PARP-1, CHK2, along with p-CHK2 with quantification. **P < 0.01 vs. Control; ^##^*P* < 0.01 vs. H_2_O_2_ + vector.

H_2_O_2_ treatment induced marked DNA fragmentation in PIG3V cells, as evidenced by increased comet tail length and intensity, both of which were significantly mitigated by MAPKAPK2 overexpression (*P*<*0.01*; Figures 5A–C). Consistently, levels of 8-OHdG, a surrogate biomarker for oxidative DNA lesions, were elevated upon H_2_O_2_ and reduced by MAPKAPK2 overexpression (*P*<*0.01*; Figure 5D). To further explore DDR signaling, Western blot analysis was performed. H_2_O_2_ exposure upregulated γ-H2AX, PARP-1, and p-CHK2 (*P* < 0.01), while total CHK2 levels remained unchanged. Notably, MAPKAPK2 overexpression suppressed the expression of γ-H2AX, PARP-1, and p-CHK2 (*P* < 0.01; Figure 5E), suggesting attenuation of DDR activation elicited by oxidative insult.

These results demonstrated that MAPKAPK2 alleviated oxidative DNA damage and was associated with reduced activation of canonical DDR markers, implying a genome-protective role in vitiligo melanocytes under oxidative stress.

### 3.6 MAPKAPK2 overexpression modulates the KEAP1/Nrf2/HO-1 axis under oxidative stress

To elucidate the molecular mechanism underlying MAPKAPK2-mediated antioxidant protection, we examined the KEAP1/Nrf2/HO-1 regulatory axis in PIG3V cells upon H_2_O_2_-induced oxidative stress.

**Figure 6:**
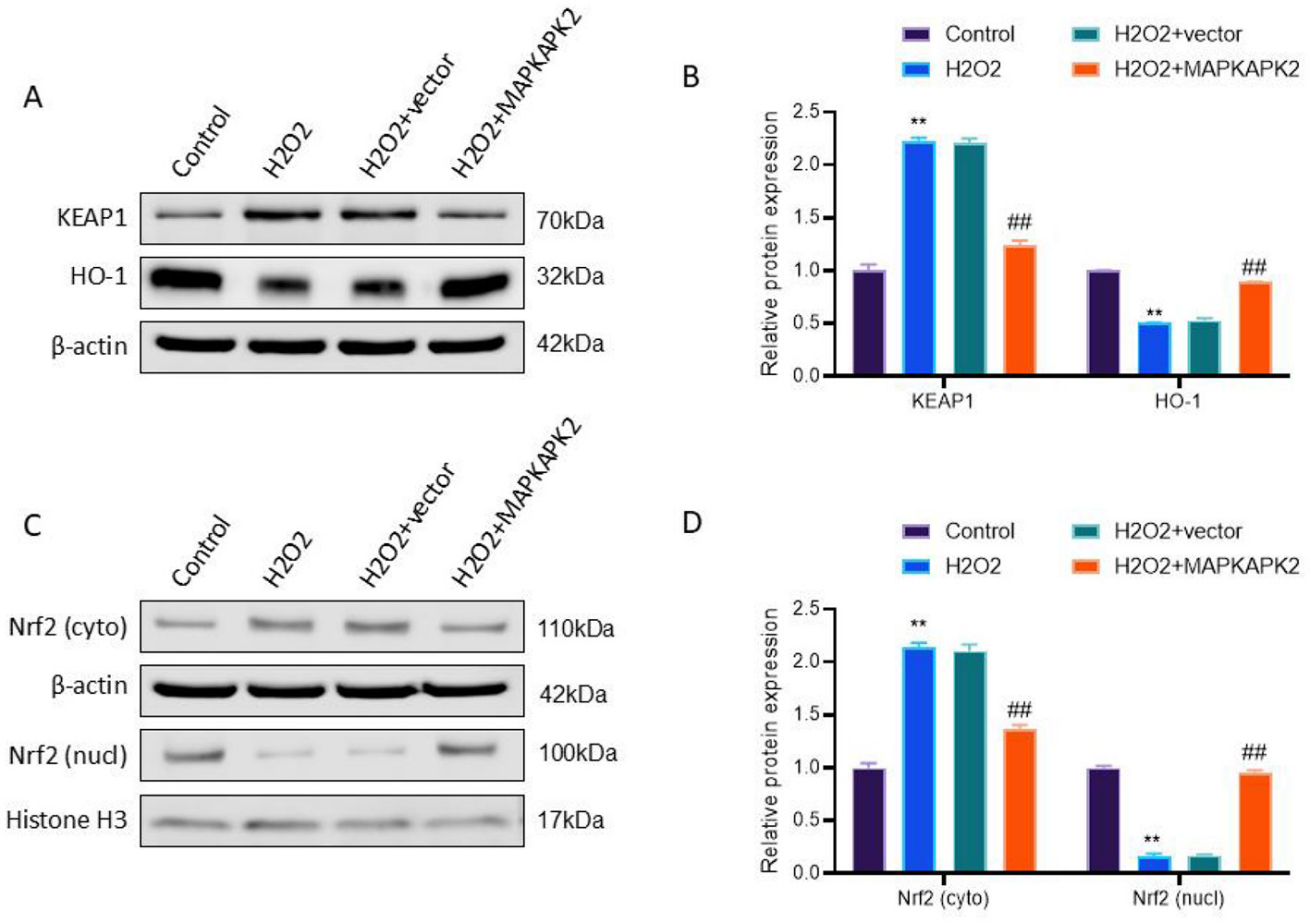
MAPKAPK2 overexpression modulates the KEAP1/Nrf2/HO-1 axis under oxidative stress. **(A, B)** Immunoblotting as well as quantification for KEAP1 and HO-1 protein expression. **(C, D)** Cytoplasmic and nuclear Nrf2 protein levels and quantification. **P < 0.01 vs. Control; ^##^*P* < 0.01 vs. H_2_O_2_ + vector.

As shown in Figures 6A, B, H_2_O_2_ exposure triggered a notable upregulation in KEAP1 and suppression of HO-1 protein levels (*P* < 0.01). These effects were effectively reversed by MAPKAPK2 overexpression (*P* < 0.01), suggesting that MAPKAPK2 might alleviate KEAP1-mediated suppression of antioxidant responses. To assess Nrf2 activity, subcellular fractionation was performed. Oxidative stress caused increased cytoplasmic retention and decreased nuclear localization of Nrf2 (*P* < 0.01), indicating inhibition of its transcriptional function. In contrast, MAPKAPK2 overexpression promoted Nrf2 nuclear translocation, as evidenced by reduced cytoplasmic and increased nuclear Nrf2 levels (*P* < 0.01) (Figures 6C, D).

Our observations implied that MAPKAPK2 was associated with reduced KEAP1 levels and enhanced Nrf2 nuclear localization, concomitant with increased HO-1 expression, suggesting a potential role in reinforcing antioxidant defenses in vitiligo melanocytes.

### 3.7 MAPKAPK2-dependent cytoprotection under oxidative stress conditions

**Figure 7:**
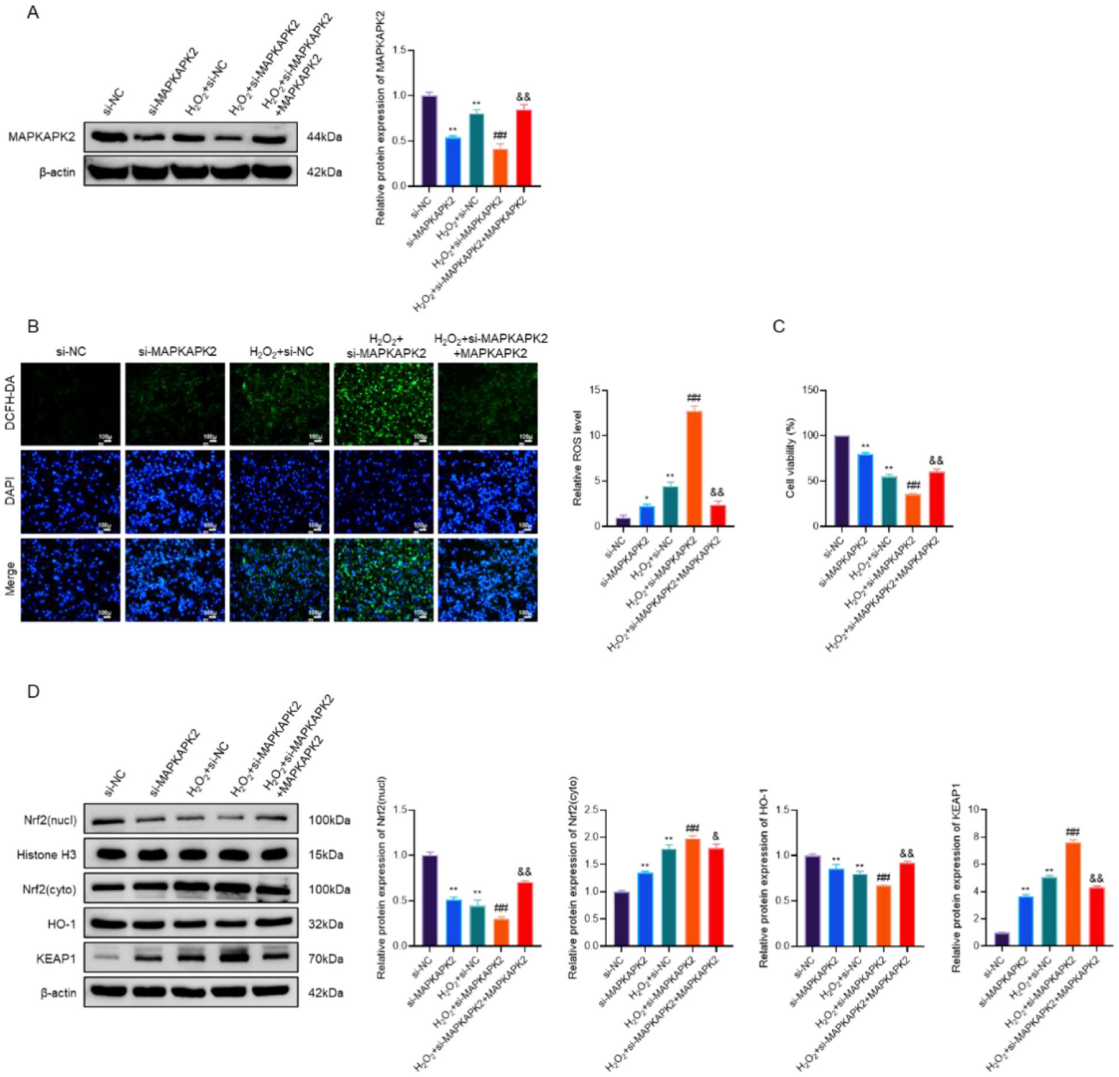
MAPKAPK2-dependent cytoprotective effects in H_2_O_2_-treated vitiligo melanocytes. **(A)** Representative immunoblot and quantitative analysis of MAPKAPK2 protein expression in PIG3V cells. **(B)** ROS levels in PIG3V cells subjected to MAPKAPK2 knockdown and rescue under H_2_O_2_ exposure. **(C)** Cell viability of PIG3V cells. **(D)** Immunoblot analysis and quantification of Nrf2 in nuclear fractions and HO-1 and KEAP1 in whole-cell lysates. *P < 0.05, **P < 0.01 vs. si-NC; ^##^*P* < 0.01 vs. H_2_O_2_ + si-NC; ^&^*P* < 0.05; ^&&^*P* < 0.01 vs. H_2_O_2_ + si-MAPKAPK2.

To further determine whether the cytoprotective effects observed upon MAPKAPK2 overexpression under oxidative stress conditions are specifically dependent on MAPKAPK2, a loss-of-function and rescue strategy was employed in PIG3V cells. As shown in Figure 7A, MAPKAPK2 protein levels were markedly reduced by si-MAPKAPK2 transfection, whereas re-expression of MAPKAPK2 effectively restored its protein abundance, confirming the efficiency of both knockdown and rescue.

Functionally, MAPKAPK2 depletion significantly exacerbated H_2_O_2_-induced oxidative stress, as evidenced by increased intracellular ROS accumulation, with ROS levels increasing by approximately 2–3 fold compared with the control group. In contrast, re-expression of MAPKAPK2 substantially attenuated ROS levels in H_2_O_2_-treated cells, reducing ROS accumulation by approximately 40%−50% relative to the H_2_O_2_ + si-NC group (Figure 7B). Consistent with these findings, cell viability assays demonstrated that MAPKAPK2 knockdown further reduced the viability of PIG3V cells exposed to H_2_O_2_, whereas restoration of MAPKAPK2 expression partially rescued cell viability under the same conditions (Figure 7C). These results indicate that the protective effects against oxidative injury are largely dependent on MAPKAPK2 expression.

To further explore the downstream antioxidant signaling associated with MAPKAPK2-dependent cytoprotection, the status of the KEAP1/Nrf2/ HO-1 pathway was examined. MAPKAPK2 knockdown impaired Nrf2 nuclear accumulation and reduced HO-1 expression while increasing KEAP1 levels in H_2_O_2_-treated cells. In contrast, re-expression of MAPKAPK2 reversed these alterations, restoring Nrf2 nuclear localization and enhancing HO-1 expression (Figure 7D), consistent with activation of an antioxidant defense response.

Collectively, these results demonstrate that the cytoprotective effects observed in MAPKAPK2-overexpressing vitiligo melanocytes under oxidative stress conditions are specifically dependent on MAPKAPK2. Genetic depletion of MAPKAPK2 abolishes these protective effects, whereas re-expression of MAPKAPK2 restores resistance to oxidative stress–induced cellular injury.

## 4 Discussion

This research assessed the MAPKAPK2-mediated regulation of oxidative stress response in vitiligo melanocytes, with particular attention to its regulatory expression pattern, functional consequences, and associated redox signaling pathways. Our results demonstrate that MAPKAPK2 expression is significantly reduced in vitiligo-derived melanocytes (PIG3V), further suppressed upon H_2_O_2_ exposure, and partially reinstated through exposure to the demethylating agent 5-aza-DC. Functionally, MAPKAPK2 overexpression mitigated oxidative damage, restored antioxidant capacity, and attenuated markers of DNA damage and cellular dysfunction, suggesting a cytoprotective role under oxidative conditions. These findings shed light on a previously uncharacterized mechanism of melanocyte vulnerability in vitiligo and identify MAPKAPK2 as a novel target of interest.

Epigenetic mechanisms are increasingly recognized as modulators of stress-responsive genes in chronic inflammatory and degenerative skin diseases (19). In our study, the observation that MAPKAPK2 expression is suppressed in PIG3V cells prompted the hypothesis that epigenetic mechanisms may contribute to its basal repression. In contrast, the further decrease observed upon H_2_O_2_ stimulation suggests an additional oxidative stress–associated regulatory component, the precise molecular basis of which remains to be elucidated. Computational analysis identified CpG islands within the MAPKAPK2 promoter, and treatment with 5-aza-DC partially rescued MAPKAPK2 mRNA and protein levels. While this suggests that DNA methylation may contribute to MAPKAPK2 repression, definitive confirmation—such as bisulfite sequencing or methylation-specific PCR—remains to be performed. Nonetheless, this finding supports a model in which oxidative stress and epigenetic silencing synergistically impair protective kinase pathways in vitiligo melanocytes.

Functionally, MAPKAPK2 overexpression significantly ameliorated H_2_O_2_-induced phenotypes, including reduced viability, increased apoptosis, impaired melanogenesis, and elevated ROS levels accompanied by reduced antioxidant capacity (GSH and SOD). The experimental evidence demonstrates that MAPKAPK2 contributes to the counteracting oxidative stress–induced damage in melanocytes. This is consistent with previous studies showing that MAPKAPK2 regulates apoptosis, inflammation, and oxidative stress (20). Our findings extend this functional relevance to melanocyte biology for the first time, highlighting MAPKAPK2′s contribution to redox homeostasis and melanocyte survival under oxidative conditions—mechanisms critically implicated in vitiligo pathogenesis (21, 22). Importantly, the loss-of-function and rescue experiments further strengthened the causal role of MAPKAPK2 in mediating cytoprotection under oxidative stress. Genetic depletion of MAPKAPK2 abolished the protective effects against ROS accumulation and cell viability loss, whereas re-expression of MAPKAPK2 restored cellular resistance to oxidative injury. These findings indicate that MAPKAPK2 is required for melanocyte resistance to oxidative stress, but do not alone distinguish whether its protective role depends on protein abundance or kinase activity.

Notably, beyond genetic manipulation, pharmacological inhibition experiments provided critical functional validation of MAPKAPK2 activity. Although selective MK2 inhibition alone did not significantly affect basal cell viability, it effectively abolished the cytoprotective effects conferred by MAPKAPK2 overexpression under oxidative stress conditions. Moreover, suppression of HSP27 phosphorylation confirmed effective blockade of MK2 kinase activity. These findings demonstrate that MAPKAPK2-mediated cytoprotection is dependent on its kinase activity rather than changes in expression alone, and that MK2 activity becomes functionally essential under oxidative challenge rather than during baseline melanocyte maintenance.

Furthermore, MAPKAPK2 overexpression reduced levels of oxidative DNA damage, as evidenced by decreased comet tail moments and lower 8-OHdG content. Concurrently, markers of DNA damage response activation, including γ-H2AX, PARP-1, and phosphorylated CHK2, were significantly downregulated. The observed outcomes suggest that MAPKAPK2 might be involved in the maintenance of genomic integrity under stress conditions (23). While these effects are likely secondary to reduced ROS burden, the possibility that MAPKAPK2 also modulates DNA repair machinery cannot be excluded and warrants further mechanistic investigation.

At the molecular level, we investigated the potential engagement of the KEAP1/Nrf2/HO-1 antioxidant axis, a key signaling cascade in maintaining redox balance (22). MAPKAPK2 overexpression was associated with downregulation of KEAP1, increased Nrf2 nuclear accumulation, and elevated HO-1 protein levels. Given that Nrf2 activity is tightly regulated by KEAP1-mediated cytoplasmic sequestration and proteasomal degradation, the observed changes suggest that MAPKAPK2 may enhance Nrf2 signaling indirectly by relieving KEAP1 inhibition (24, 25). However, the precise mechanism by which MAPKAPK2 influences this axis—whether transcriptionally, post-translationally, or via upstream kinase pathways—remains to be elucidated. Future studies using Nrf2 knockdown, KEAP1 overexpression, or reporter assays are needed to confirm the specificity and directionality of this regulatory interaction.

Although this study first validates the role of MAPKAPK2 in melanocytes, several limitations remain. First, all experiments were conducted *in vitro* using immortalized melanocyte cell lines (PIG1 and PIG3V). While these models provide reproducible systems for mechanistic investigation, immortalization may introduce molecular alterations that differ from the *in vivo* disease context. Therefore, future studies should validate key observations—particularly MAPKAPK2 kinase activity and expression patterns—in primary melanocytes or lesional skin samples from vitiligo patients to enhance clinical relevance. Second, oxidative stress was modeled using a single concentration of H_2_O_2_. Although this approach reliably induces oxidative injury *in vitro*, it may not fully reflect the chronic and multifactorial oxidative environment of vitiligo. Future research should include dose–response analyses or employ alternative stressors, such as UV irradiation or IFN-γ exposure, to better simulate *in vivo* conditions. Third, the molecular mechanisms underlying MAPKAPK2 regulation require further elucidation. The direct interaction between MAPKAPK2 and Nrf2 remains to be confirmed through luciferase reporter or co-immunoprecipitation assays, and the association between MAPKAPK2 expression and promoter methylation should be validated using bisulfite sequencing or methylation array analyses. Finally, although intracellular GSH levels were measured in the present study, the expression of glutathione synthesis–related enzymes was not examined. Future studies should further investigate this downstream antioxidant program to better clarify the involvement of glutathione metabolism in MAPKAPK2-mediated regulation of the KEAP1/Nrf2 pathway.

In summary, our findings identify MAPKAPK2 as a potentially epigenetically silenced, stress-responsive kinase that mitigates oxidative damage and supports antioxidant defense in vitiligo melanocytes. Importantly, intact MAPKAPK2 kinase activity is required for these protective effects under oxidative stress conditions. Through modulation of redox signaling pathways and attenuation of DNA damage responses, MAPKAPK2 emerges as a novel molecular candidate with potential relevance for future therapeutic strategies targeting oxidative stress in vitiligo.

## 5 Conclusion

MAPKAPK2 is markedly downregulated in vitiligo melanocytes, likely due in part to epigenetic repression, which may render melanocytes more susceptible to oxidative stress–induced damage. Overexpression of MAPKAPK2 effectively alleviated key pathological features, including ROS accumulation, impaired melanogenesis, and increased apoptosis, potentially via KEAP1/Nrf2/HO-1 signaling activation. Importantly, genetic loss-of-function, rescue, and pharmacological inhibition analyses further demonstrated that intact MAPKAPK2 kinase activity, rather than protein expression alone, is indispensable for maintaining melanocyte resistance to oxidative stress. These findings highlight the regulatory role of MAPKAPK2 in the oxidative stress response of vitiligo melanocytes and underscore its functional relevance in vitiligo pathogenesis, providing a rationale for future targeted therapies.

## Data Availability

The datasets presented in this study can be found in online repositories. The names of the repository/repositories and accession number(s) can be found in the article/Supplementary material.
