# Supplementary material for: Epigenetic downregulation of MAPKAPK2 exacerbates oxidative stress-induced damage in vitiligo melanocyte cell line model
Source: Front Med (Lausanne). 2026 Apr 20;13:1698091. doi: 10.3389/fmed.2026.1698091 (PMC13136003; doi:10.3389/fmed.2026.1698091)
Supplement: Supplementary file 1 [file Supplementary_file_1.docx]

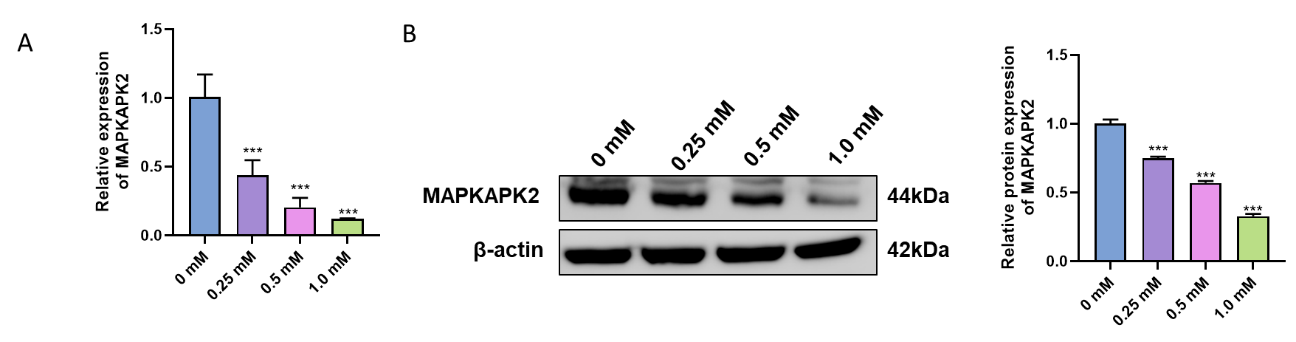


Figure S1. Dose-dependent suppression of MAPKAPK2 expression by H₂O₂ in vitiligo melanocytes.

(A) Relative MAPKAPK2 mRNA expression in PIG3V cells treated with increasing concentrations of H₂O₂ (0, 0.25, 0.5, and 1.0 mM), as determined by qRT-PCR. (B) Representative immunoblot and corresponding densitometric analysis showing MAPKAPK2 protein expression in PIG3V cells following H₂O₂ treatment at the indicated concentrations. ***P < 0.001 vs. Control (0 mM).


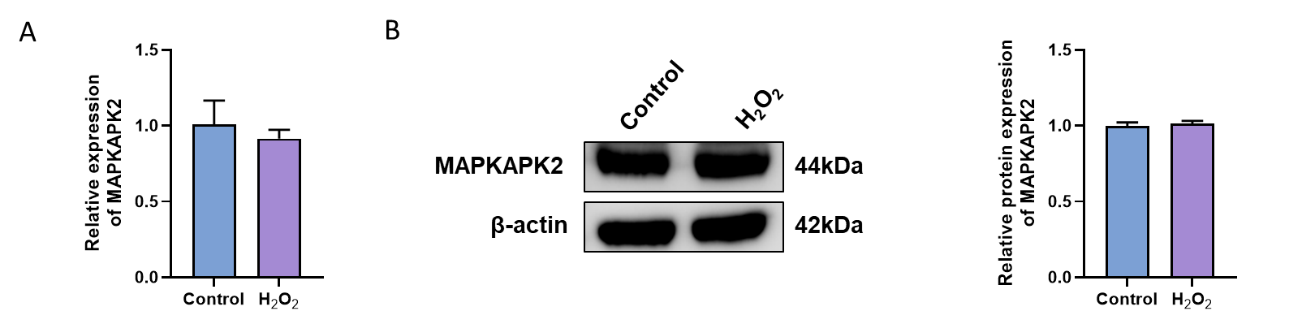


Figure S2. Effect of H₂O₂ on MAPKAPK2 expression in non-vitiligo melanocytes (PIG1).

(A) Relative MAPKAPK2 mRNA expression in PIG1 cells treated with H₂O₂ (1.0 mM) for 24 h, as determined by qRT-PCR.

(B) Representative immunoblot and densitometric analysis of MAPKAPK2 protein expression in PIG1 cells following H₂O₂ treatment.
